# A community health volunteer delivered problem-solving therapy mobile application based on the Friendship Bench ‘Inuka Coaching’ in Kenya: A pilot cohort study

**DOI:** 10.1017/gmh.2021.3

**Published:** 2021-03-10

**Authors:** Asmae Doukani, Robin van Dalen, Hristo Valev, Annie Njenga, Francesco Sera, Dixon Chibanda

**Affiliations:** 1 Department of Population Health, London School of Hygiene & Tropical Medicine, Keppel Street, London WC1E 7HT, UK; 2 Inuka Foundation, Rapsodieplantsoen 11, 1312EJ Almere, Netherlands; 3 Philips Research, High Tech Campus 34, 5656 AE Eindhoven, Netherlands; 4 Department of Statistics, Computer Science and Applications “G. Parenti”, University of Florence, Florence, Italy

**Keywords:** Community health workers, sub-Saharan Africa, e-mental health, Friendship Bench, problem-solving therapy

## Abstract

**Background:**

Sub-Saharan Africa (SSA) has the largest care gap for common mental disorders (CMDs) globally, heralding the use of cost-cutting approaches such as task-shifting and digital technologies as viable approaches for expanding the mental health workforce. This study aims to evaluate the effectiveness of a problem-solving therapy (PST) intervention that is delivered by community health volunteers (CHVs) through a mobile application called ‘Inuka coaching’ in Kenya.

**Methods:**

A pilot prospective cohort study recruited participants from 18 health centres in Kenya. People who self-screened were eligible if they scored 8 or higher on the Self-Reporting Questionnaire-20 (SRQ-20), were aged 18 years or older, conversant in written and spoken English, and familiar with the use of smart mobile devices. The intervention consisted of four PST mobile application chat-sessions delivered by CHVs. CMD measures were administered at baseline, 4-weeks (post-treatment), and at 3-months follow-up assessment.

**Results:**

In all, 80 participants consented to the study, of which 60 participants (female, *n* = 38; male, *n* = 22) completed their 4-week assessments, and 52 participants completed their 3-month follow-up assessment. The results showed a significant improvement over time on the Self-Reporting Questionnaire-20 (SRQ-20). Higher-range income, not reporting suicidal ideation, being aged over 30 years, and being male were associated with higher CMD symptom reduction.

**Conclusion:**

To our knowledge, this report is the first to pilot a PST intervention that is delivered by CHVs through a locally developed mobile application in Kenya, to which clinically meaningful improvements were found. However, a randomised-controlled trial is required to robustly evaluate this intervention.

## Introduction

Mental health conditions affect the wellbeing of hundreds of millions of individuals worldwide, causing considerable disability and incurring high economic and social costs (Patel *et al*., 2013). Low- and middle-income countries are disproportionately affected by the burden of mental health conditions, with sub-Saharan Africa (SSA) experiencing the largest care gap for common mental disorders (CMDs). While one in four people suffer from depression and anxiety every year on the African continent (Walker *et al*., 2015), up to 90% of people in SSA will not receive the mental health care they require due to mostly underdeveloped and poorly resourced mental health services, inadequate availability of mental health professionals, and as a consequence of the stigma associated with seeking treatment for mental health conditions (Bruckner *et al*., 2011; Becker and Kleinman, 2013; Naslund *et al*., 2017).

In 2011, the World Health Organisation (WHO) projected a shortage of 1.71 million mental health workers in low- and middle-income countries, requiring an estimated 6.4 billion US dollars to bridge the gap in human resources (World Health Organization, 2011). For this reason, WHO have heralded task-shifting as a keystone approach for increasing the human workforce in low resource settings. Task-shifting refers to the training of staff that are typically lower skilled and or have lower qualifications, to undertake activities that are either generally performed by higher qualified professionals or which they have not carried out before (WHO, 2008). The task shifting approach is not only used to shift tasks from higher to lower-cadre staff, but also to individuals living in the community, commonly referred to as non-specialist workers.

In the past decade, the evidence-base for cost-effective interventions for mental health conditions in low- and middle-income countries has steadily increased (van Ginneken *et al*., 2013). The literature on non-specialist worker delievered self-help training have found these interventions to be helpful in reducing symptoms of trauma, depression and anxiety disorders in low- and middle-income countries, including countries in SSA. A prime example is the Friendship Bench in Zimbabwe (Chibanda *et al*., 2016), in which community grandmothers where trained and supervised to deliver a problem-solving therapy (PST) intervention to treat CMDs. A randomised-controlled trial evaluating the Friendship Bench in 24 clinics in Harare found statistically significant improvements in depression, anxiety, quality of life and functioning among the 246 participants in the intervention arm, when compared to the control arm (Chibanda *et al*., 2016).

While task-shifting is championed for strengthening and expanding the workforce (Shah, 2011; Seidman and Atun, 2017), the use of technological innovations such as mobile devices and web-based platforms hold promise in reducing mental health care access barriers associated with stigma, cost of care and quality of service provision (Patel *et al*., 2013). For example, the integration of digital technologies in mental health care may reduce the costs of delivering and accessing services, while also extending access in relation to time and location (Andersson and Titov, 2014; Knowles *et al*., 2014).

Technological capacity is fast increasing in the African continent. The SSA region has the fastest growing mobile phone use and coverage in the world, in which 444 million unique mobile subscribers and 250 million smartphone connections were registered at the end of 2014 (GSMA, 2018). The spread of inexpensive smartphones is likely to push internet penetration in the region to 50% within a decade (The Economist, 2015). However, while the evidence for digital mental health interventions is gaining momentum globally, very few of these studies were conducted in low-income countries (Ndetei *et al*., 2007; Anthes, 2016; Abaza and Marschollek, 2017). A study comparing the literature on mobile health between high- and low- and middle-income countries found that while short message service solutions were prevalent across all country-income levels, mobile-app solutions were mostly used in high-income countries (Abaza and Marschollek, 2017). A narrative review on the use of digital technologies for treating and preventing mental health conditions in low- and middle-income countries found that the evidence largely came from acceptability and feasibility studies showing the potential effectiveness of online, text-messaging, and telephone support interventions. Out of the 49 studies identified in the systematic review, 14% (*n* = 7) were from Africa, compared to Latin America (19; 29%), and south Asia (5; 10%). Moreover, the studies based in Africa primarily focused on people with serious mental health conditions, with none centred on preventive interventions (Naslund *et al*., 2017).

Considering the dearth of research on preventive digital mental health interventions in low- and middle-income countries, our study aims to evaluate the effectiveness of a PST intervention for CMDs, that is delivered by community health workers (CHVs) through a mobile application (app) in Kenya. To our knowledge, our study is the first to undertake this line of research in the SSA region. The objectives of the study are to carry out a prospective cohort study to understand: (a) the acceptability of the intervention by clients, (b) evaluate the difference in self-reported CMDs before and after the intervention, and (c) to understand if sociodemographic variables predict improvements in CMD symptoms.

## Methods

### Setting and participants

A pilot prospective cohort study was carried out to evaluate a CHV-delivered PST mobile app intervention for people experiencing CMDs. In the second quarter of 2017, participants were recruited from 18, level III health facilities^1^ in primary care services in the county of Ruiru, Kenya which covers a catchment population of 25 000. Ethical clearance was approved by the ICBE Secretary on behalf of the ICBE Board (2015-0032) on 14th February 2017 and the Amref Ethics and Scientific Review Committee on 10th April 2017 (KIAMBU/HRDU/AUTHO/2017/04/10/Njenga A). The recruitment of participants was proceeded by a mental health campaign to promote knowledge around mental health and wellness and about the pilot study within the community and in level-III health facilities. The campaign involved three approaches. First, flyers were distributed to all patients and family members who visited the health centre. Second, healthcare providers in the centre were encouraged to talk about mental health and wellness and propose the study to all clients seeking preventive or curative health services. Third, CHVs who were not involved in the delivery of the intervention, were encouraged to talk about mental health and wellness, and psychological support during the routine ‘dialogue days’ held in the community. Potential participants were approached about the study at the health centre and at home by healthcare providers and members of the research team. Those interested in participating in the study were invited to self-screen at the health centre.

### Inclusion criteria

The study included people who, scored 8 or higher on the Self-Reporting Questionnaire-20 (SRQ-20) (World Health Organization, 1994), were aged 18 years or older, were conversant in written and spoken English, familiar and comfortable with the use of smart mobile devices (phone/tablet/touch-based screens), and were willing to attend the health facility for the intervention. People who attended the health centre to self-screen for the study were recompensed with a transport allowance of KSh 300 (the equivalent of £2.17 GBP in 2017). Participants who met the criteria and were willing to participate in the study provided written informed consent with the assistance of health professionals at the health centre. Participants were then asked to indicate their availability on a calendar to confirm their schedule for the four-week programme.

### Community Health Volunteers

CHVs working in the Community Health Strategy Unit, in the rural constituency of Kiu in Nairobi were recruited as ‘coaches’ on the programme. CHVs were appointed if they were able to speak and write English and if they owned a smart phone and regularly used it for communication.^2^ Those who did not meet the criteria were excluded. From an existing pool of 15 CHVs community extension workers identified five suitable CHVs who were invited to a three-day training programme in the second quarter of 2017. CHVs were recompensed KSh 300 (the equivalent of around £2.17 GBD in 2017) per training session, and per coaching session on the programme. CHVs were trained to coach participants through the PST intervention on the Inuka mobile app by a senior training specialist who worked on the Friendship Bench (Chibanda *et al*., 2016). The three-day training took place in the health centre and consisted of an introduction to PST (day 1), a step-by-step guide to PST (day 2), and mock practice session on delivering PST (day 3). CHVs were provided with standard text material to guide their interactions with the client on the app, and where encouraged to practice to ensure that their communication was spontaneous and steeped in contextual information provided by the client.

All five CHVs recruited were, female, aged between 33 and 42 years and at minimum had a secondary school education. One of the CHVs fell ill at the start of the pilot study and was unable to continue delivering the intervention. The remaining four CHVs continued their involvement until the end of the project. At the end of each day of the programme, a group session which included CHVs, a psychologist and a member of the research team was arranged to understand the CHVs experience of delivering the intervention and for supervision purposes. The outputs from these meetings were not documented systematically (e.g. survey, qualitative interviews etc.). During the course of the study, each CHV received two one-to-one sessions with a psychologist who provided them with personal mental health support.

### Inuka coaching

‘Inuka’, is a Swahili word meaning ‘arise’. The Inuka coaching programme consisted of structured PST sessions that were delivered to clients through a text-based chat on a mobile-app. The intervention follows the structure of a low-intensity face-to-face PST intervention, which has in recent years been adapted to be delivered by non-specialist workers for the treatment of CMDs across a range of populations in the African and Asian continent (van Ginneken *et al*., 2013; Sijbrandij *et al*., 2016). The PST intervention was identified as a suitable approach for delivering via non-traditional formats such as telephone, due to it's simple step-by-step approach (Hoek *et al*., 2012).

The intervention consisted of four chat-sessions that were delivered through a chat-based service on an android device (Techno-M6S Android mobile version 4.4.2 KitKat/5.0 inches/Dual-Sim/8 GB) in the health centre. A research assistant called participants to remind them of their appointment ahead of their session. Participants attended weekly or bi-weekly sessions at the health centre which were expected to last up to 60 minutes each. Participants were recompensed KSh 300 for travelling to the health centre. Participants completed their sessions in the health centre for three reasons. First, it was unclear if participants had access to a mobile device or the internet. Delivering the intervention in the health centre was therefore anticipated to reduce barriers to participation in the study. Second, holding the session in the health centre enabled participants to receive help in using the mobile app and in resolving technical issues. Access to help was especially important given that the Inuka coaching app was still in the Beta phase (a complete app that may contain glitches). Third, not enough funds were available to allocate an android phone and a data plan to each participant recruited to the study.

CHVs delivered the intervention in the clinic, from a different part of the health centre to prevent meeting participants face-to-face, to ensure their identity stayed anonymous. During the start of the project it emerged that the CHVs' personal mobile phones were not suitable for use in the study for several reasons. First, CHVs often used their phones for other purposes (e.g. personal, other work responsibilities etc.) which at times disrupted their sessions (e.g. incoming calls, messages). Second, their android phones often did not have enough battery-life to complete a typical session that was expected to last up to 60 mins. Moreover, the design of their mobile phones (e.g. screen, keypad) varied significantly and were not always suitable for delivering a chat-based service, leading to typographical errors. As a result, all coaches were provided with a standard android phone (Techno-M6S Android mobile version 4.4.2 KitKat/5.0 inches/Dual-Sim/8 GB), which they accessed at the health centre to ensure that a power supply was available. It was also discovered that CHVs heavily relied on standard text-scripts or material to deliver the intervention. Additional time to practice was therefore arranged in group supervision meetings.

The Inuka coaching intervention had four key features, (1) matching the client with the coach, (2) screening and support, (3) decision support for CHVs, (4) and promotion of psychological self-care, described in Table 1. The role of the CHV involved providing participants with the tools to effectively identify and solve problems that arise in their life, to reduce psychological distress and enhance wellbeing. Coaches were required to follow the seven-step procedure outlined below.

**Table 1.** A summary of the five principles of the Inuka coaching intervention.

Principle	Descriptions
Matching	Person seeking psychological support (client) is matched with a service provider.
Screening and support	The client is screened for CMDs using the SRQ-20 and is engaged in a structured conversation based on PST via a text-based chat with the service-provider.
Decision support for CHVs	The service-provider is provided with a set of structured scripts on what to say in each step of the intervention and how to build rapport via text-based chat. The service provider fills out an action plan which summarises the clients problems and next steps.
Referral	If the service provided is deemed insufficient by the client or if the service provider deems the client's presentation as severe, the client is referred to a mental health professional for diagnosis and treatment with their consent.
Promotion of psychological (self) care	The Inuka coaching app provdes information and self-assessment services that are contextualised for local settings to lower the barriers for seeking help.

1. Problem list

The coach helps the client identify a set of problems that are negatively impacting their mental health. Through a process of empathic listening, coaches attempted to gain an understanding of the client's problems across a range of different domains (e.g. work, wellbeing, relationships, financial, physical or spiritual). The coach demonstrates empathic and attentive listening through asking clarifying questions, validating the client's experiences, and through summarising the client's problems under a list called ‘things to tackle’. Step 1 is generally carried out in the first session, however, the list can be amended in follow-up sessions if and when new information comes to light.

1. Selecting a problem

The coach assists the client in selecting one problem to address in the session, through asking questions such as, ‘which problem is the most important for you to resolve?’ The client is also encouraged to evaluate the feasibility of directly solving the problem (e.g. through assessing how much control they have over a given situation or problem).

1. Goal setting

The coach helps the client to set a goal in relation to their selected problem, through asking questions such as, ‘how would your life look like if you managed to solve this problem?’ and through provding guidance on how to set goals that are specific, measurable, attainable, relevant and time-based (SMART).

1. Brainstorming for solutions

The coach will then help the client to brainstorm solutions to their problem.

1. Selecting a solution

The coach assists the client to assess the suitability of the solutions that they have brainstormed (e.g. exploring the pros and cons of each solution or listing their solutions in order of preference) and through empowering the client to select a solution to take forward and implement.

1. SMART action plan

The coach then guides the client in developing a SMART action plan. During the formulation of the action plan, the coach will also help the client to think through possible barriers to implementing their plan, and how such barriers can be overcome. The session ends by reviewing steps 1−6, and by providing the client with a wellbeing plan.

1. Evaluation of the action plan

The final step involves evaluating the success of the action plan, which typically takes place in the second session. If the action plan was effective in addressing the client's problem, the coach proceeds to guide the client through another problem. If the solution was ineffective, the coach will use the same session to explore with the client why the solution was ineffective. The coach and the client will then either revise or develop a new action plan.

### Design and development of Inuka coaching

The design, development and evaluation of Inuka coaching was undertaken through a close collaborations between the commercial and academic sectors. A working prototype of the platform was developed and tested between 2015 and 2016 by Philips in collaboration with the University of Zimbabwe. The design, development and testing of the Inuka app will be outlined in another paper. The pilot study data was collected while the platform was owned by Philips. Currently, the Inuka platform is independent of Philips and is a social enterprise start-up owned by author RvD (inuka.io). Informal academic collaborations have been extended to the London School of Hygiene and Tropical Medicine. At present, the Inuka platform is a guided self-help mental health prevention intervention with therapeutic effects, and is not a medical device.

### Measures

Participants were assessed at baseline, at the end of the intervention (4 weeks), and at the 3-months follow-up assessment.

### Primary outcome

#### CMD symptoms

The self-reporting questionnaire-20 (SRQ-20) is a 20-item self-report screening tool that was developed by the World Health Organisation (World Health Organization, 1994). Questionnaire items are responded to through a dichotomous yes/no response options to indicate if symptoms across four scales; anxiety and depression, somatic symptoms, reduced vital energy, and depressive thoughts,were present or absent in the past month. All yes responses were totalled to produce a composite score. Higher scores indicate a higher presence of symptoms. The SRQ-20 has been validated in clinical populations in Kenya (Dhadphale *et al*., 1983). The questionnaire was primarily developed for use in primary health care settings, especially in low- and middle-income countries. The SRQ-20 was administered at baseline, during the last session of the intervention (4 weeks) and at the 3-months follow-up assessment.

### Secondary outcome

#### Depression

The Patient Health Questionnaire (PHQ-9) (Kroenke *et al*., 2001) is a 9-item self-report scale that assesses the presence of symptomatic criteria of a major depressive episode and the severity of each symptom over the last two weeks. The frequency of each symptom is evaluated through a 4-point Likert scale, from 0 (not at all) to 3 (nearly every day). Total scores range from 0 to 27 with higher scores indicating greater depression severity. The PHQ-9 has been shown to have good psychometric properties (Wittkampf *et al*., 2000; Kroenke *et al*., 2001). The PHQ-9 was administered at baseline, post treatment (4 weeks) and at the 3-months follow-up assessment (Monahan *et al*., 2009).

#### Anxiety

GAD-7 (Spitzer *et al*., 2006) is a 7-item self-report scale used to screen for Generalised Anxiety Disorder in primary care settings. Each items is scored on a four-point scale (from zero to three) with total scores ranging from 0 and 21 and higher scores reflecting greater anxiety severity. The cut-off scores for mild, moderate and severe anxiety symptoms are 5, 10 and 15, respectively. The GAD-7 was validated in the SSA region (Chibanda *et al*., 2016). The Internal consistency of the scale was estimated at 0.92 and convergent validity was established by means of correlations with two other anxiety measures (Spitzer *et al*., 2006). The GAD-7 was administered at baseline, post-treatment (4 weeks) and at the 3-months follow-up assessment.

### Other forms

#### Sociodemographic

Demographic data on gender, age (captured through five age brackets), educational attainment and income were collected at baseline assessments.**^3^**

#### Trauma checklist

The Confounders questionnaire is a checklist of nine potentially traumatic events experienced in the past 30 days (e.g. ‘Have you lost your loved one') which are responded to with a dichotimous ‘Yes’ or ‘No’ response option. The number of ‘yes responses’ were tallied for each participant and per item on the questionnaire. The questionnaire was administered at baseline assessments.

#### Session and programme rating questionnaires

The session and programme rating questionnaire is an 8-item self-report scale measuring satisfaction with the Inuka coaching intervention. Items were rated by the participant on a scale of 1–4 with total scores ranged between 8 and 23. Satisfaction with the intervention were indicated on separate forms for the session with the CHV and the programme mobile app. Higher responses on the scale indicated greater satisfaction with the session and the programme.

### Statistical analysis

Growth models using multilevel modelling on Python (version 3.6.10) (Python Software Foundation, 2019), a programming language software, was used to analyse the continuous outcome data (SRQ-20 scores). Growth modelling was used to model change rate over time while taking into account possible non-independence of repeated measurement by incorporating random effects. Baseline variables of changes over time were modelled with an interaction term between the predictor and the dummy variable that model the time effect (baseline and 3-month follow-up assessments). Multiple linear regression models were performed on Rstudio (version 1.1.456) (RStudio Team, 2018), to investigate if age, gender, income, marital status, suicidal ideation were associated with pre- and post-therapy score changes for using the SRQ-20, PHQ-9 and GAD-7 self-reported questionnaires. A complete case analysis approach was used in the study.

## Results

### Participant characteristics

**Fig. 1.**
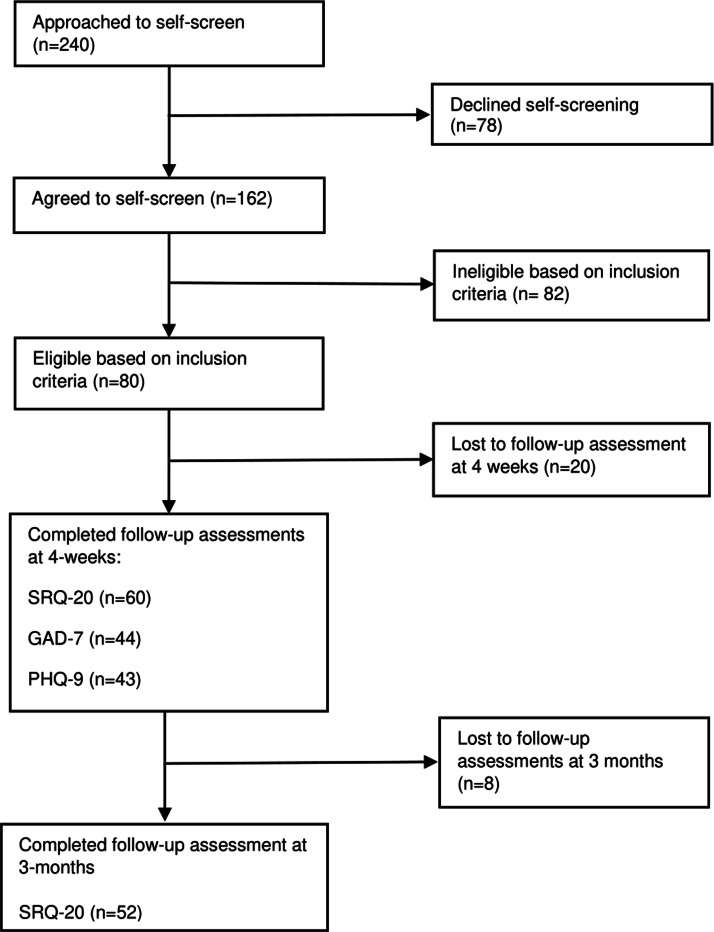
Consort flow diagram of participant recruitment and assessment in a cohort pilot study.

A total of 240 people were approached about the study, of which 67.5% (n=162) agreed to be screened and 32.5% (n=78) refused to take part in the study. The reasons for refusal included, not having working hours that were compatible with the opening hours of the health centre (9 am−4 pm); not being able to use the intervention at home; and not being comfortable or interested in engaging with the programme. The reasons for refusal were not quantified, therefore we do not know the prevalence of the reasons for refusal. Of the 162 people screened, a total of 80 people (49%) were eligible for participation, while the remaining 82 people (51%) did not meet the inclusion criteria and were therefore excluded. Of the 80 people consented and enrolled to the study 20 (25%) participants were lost to follow-up assessments at 4 weeks. 60 participants completed the SRQ-20 questionnaire, while fewer participants completed GAD-7 (*n* = 44) and PHQ-9 (*n* = 43) at 4 weeks. In total, 52 (65%) of all consenting participants completed their 3-month follow-up assessments, while eight participants (10%) were lost to the 3-months follow-up assessment. A consort flow diagram of recruitment and assessment in the study can be found in Fig. 1.

Participants were mostly female, aged between 21 and 30 years, single, secondary school educated, in paid employment, and living on an income of less than KSh. 10 000 (the equivalent of £72 GBD in 2017) per annum (p/a). The most prevalent reason attributed to seeking treatment, as reported on the Confounders questionnaire was, experiencing ‘relationship issues with family’ (50%), feeling ‘heartbroken’ (48%), and ‘losing a relative or losing a job’ (45%) (see Table 2, for full participant characteristics and a breakdown of the reasons for seeking treatment). Participants reported finding out about the study through friends, relatives, and the health professionals leading the mental health campaign in the waiting-bay of the health centres.

**Table 2.** Baseline characteristics of participants enrolled on the study (*N* = 60). Figures are numbers (percentages) of participants unless otherwise indicated.

Baseline characteristics	*N (%)*
**Gender**	
Female	*38 (63)*
Male	*22 (37)*
**Age group^a^**	
20 year old or younger	*5 (8)*
21−30 years old	*37 (62)*
31−40 years old	*12 (20)*
41−50 years old	*5 (8)*
51 years old or older	*1 (2)*
**Marital status^b^**	
Single	*33 (55)*
Married	*22 (37)*
Divorced or separated	*5 (8)*
**Level of education^c^**	
No formal education	*1 (2)*
Primary school	*9 (15)*
Secondary school	*24 (40)*
College or tertiary intuition	*21 (35)*
University degree	*5 (8)*
**Income**	
Less than KSh. 10000	*46 (77)*
More than KSh. 10000	*14 (23**)***
**Occupational status**	
Employed	*26 (43)*
Unemployed	*22 (37)*
House-wife	*7 (12)*
Student	*5 (8)*
**Suicidal^d^**	
Yes	*15 (25)*
No	*45 (75)*
**Trauma checklist^e^ (*n* = 46)**	
Lost your loved one	*13 (28)*
Lost or any of your close relative lost his/her job	*20 (43)*
Heartbroken	*22 (48)*
Relation issues with family member or relative?	*23 (50)*
Undergoing some treatment or has been ill for some time (e.g. heart failure, HIV/AIDS, diabetes)?^f^	*9 (20)*
Family member or relative undergoing some treatment or has been ill for some time (e.g. heart failure, HIV/AIDS, diabetes)?	*19 (41)*
Facing challenges with substance/drug abuse?	*2 (4)*
Family member or relative currently facing challenges with substance/drug abuse?	*17 (37)*
Experienced a traumatic event?	*17 (30)*
Other issue that has caused you distress?	*19 (41)*

Note: s.d. = standard deviation, IQR = Inter-quarter-range.

Some baseline characteristics were merged for the analysis:

a 20 years of old and younger, and 21−30 years old were merged to form a new category ‘30 years and below’ (*n* = 42). All other categories were merged to form a second ‘31 years of age or older’ category” (*n* = 18).

b Single, divorced and separated categories were merged to form a new category ‘single’ (*n* = 38), while ‘married’ stayed the same (ie. *n =* 22).

c No formal education, primary school, secondary school categories were merged to form a new category ‘secondary school or below’ (*n* = 34), while college or tertiary institution and university degree were merged to form a second category ‘college or university educated’ (*n* = 26).

d Suicidal ideation level was assessed at baseline through item 17 of the SRQ-20 questionnaire (‘thinking of ending life’) which is responded to with either a ‘yes’ indicating presence, and ‘no’ indicating absence.

e Reason for seeking treatment was assessed through the Confounder's questionnaire. Only 46 participants completed this questionnaire.

f 45 out of the 46 people who completed the Confounders questionnaire provided a response for this item.

### Session completion

In all, 60 participants completed all four sessions of Inuka coaching in which an average of 41.79 minutes was spent during each session. On average men spent 1.56 minutes longer than women. Online Supplementary Table 1 reports the average time spent in minutes in each session by gender.

### Satisfaction with the Inuka coaching session and programme

After the final session, participants rated the Inuka coaching session with the CHV and programme app on a variable scale of 1–4, with a higher number indicating greater satisfaction. The most common rating for each item was a 4 (highest rating of satisfaction), accounting for 48%–99% of all item responses. All participants indicated that they would definitely (a score of 4) or generally (a score of 3) recommend the programme to a friend. And all but one participant said that they would definitely come back to the programme if they had to seek help again (score of 4). These findings suggest that participants were highly satisfied with the CHVs delivering the intervention and the programme app. The average rating per question for the coaching session and programme are presented in online Supplementary Table 2.

### Paired samples *t* tests

A paired samples *t* test was calculated to investigate if there was a difference in mean scores between pre- and post-treatment scores on the SRQ-20, PHQ-9 and GAD-7 questionnaires. The findings indicated that there was a significant difference between pre- and post-scores on the SRQ-20, (*t*(59) = 6.94, *p* < 0.001), PHQ-9 (*t*(27) = 3.98, *p* < 0.001), and GAD-7 (*t*(27) = 3.33, *p* < 0.001). The corresponding means and standard deviations can be found in Table 3.

**Table 3.** Mean, standard deviation and range for the SRQ-20, PHQ-9 and GAD-7 for pre- and post-treatment.

	Pre-treatment		Post-treatment	
	*M*	s.d.	*M*	s.d.
SRQ-20 (*n* *=* 60)	*12.13*	*2.75*	*7.50*	*5.33*
PHQ-9 (*n* = 43)	*11.86*	*6.28*	*6.62*	*6.13*
GAD-7 (*n* = 44)	*11.89*	*4.72*	*7.00*	*6.65*

Note: s.d. = standard deviation, SRQ-20 = Self-reporting Questionnaire-20; PHQ-9=Patient Health Questionnaire-9 and GAD-7 = General Anxiety Disorder-7.

### Random effects model

A multivariate multilevel growth model was fitted using pre- and post-measurement of the SRQ-20 as the dependent variable. SRQ-20 scores decreased on average by 0.53 scores in relation to time (baseline and three-month follow-up assessment points) <0.001) (95% CI: −3.954 to −2.636), while controlling for age, gender, marital status, educational status, income and suicidal ideation.

### Baseline variables and SRQ-20 change score coefficients

A multiple linear regression investigating if age, gender, income, marital status and suicidal ideation level at baselinewere associated with SRQ-20 change scores, while adjusting for baseline SRQ-20 scores, is reported in Table 4. The model was significant, with all variables included in the model accounting for 25% of the variance in SRQ-20 change scores. Income was significantly associated with SRQ-20 scores, suggesting that participants with a higher range-income (earnings more than KSh. 10 000 p/a) experienced a higher decrease in SRQ-20 scores compared to those with a lower-range income (below KSh. 10 000 p/a). Suicidal ideation level was also significantly associated with SRQ-20 change scores, in which participants who did not express suicidal ideation experienced a higher decrease in SRQ-20 scores compared to participants who reported suicidal ideation.

**Table 4.** Summary of a multiple linear regression model for variables predicting SRQ-20, PHQ-9 and GAD-7 change scores.

	SRQ-20			PHQ-9			GAD-7		
	*B*	*s.e. B*	*CI 95*	*B*	*s.e. B*	*CI 95*	*B*	*s.e. B*	*CI 95*
Age	*−0.09*	*1.41*	*−2.676 to 2.854*	*−5.89*	*2.26*	−1.460 to 10.323*	*1*.*11*	*2*.*80*	*−4.371 to 6.590*
Gender	*−1.71*	*1.39*	*−4.440 to 1.021*	*−1.78*	*2.72*	*−7.107 to 3.538*	*−7*.*52*	*3*.*65*	−14.678 to 0.367*
Income	*−3.17*	*1.53*	−6.162 to 0.180*	*−0.37*	*2.44*	*−5.166 to 4.417*	*−5*.*68*	*3*.*13*	*−11.825 to 0.463*
Marital status	*0.43*	*1.34*	*−2.208 to 3.062*	*−0.28*	*2.14*	*−4.468 to 3.911*	*−0*.*62*	*2*.*81*	*−6.130 to 4.880*
Suicidal ideation	*4.44*	*1.46*	1.584–7.288**	*4.63*	*2.19*	0.335–8.923*	*2*.*85*	*2*.*88*	*−2.794 to 8.494*
	*R*^2^ *=* *0.25; F* for change in *R*^2^ *=* 2.87*			*R*^2^ *=* *0.57; F* for change in *R*^2^ *=* 4.57**			*R*^2^ *=* *0.46; F* for change in *R*^2^ *=* 2.98*		

Note: Age was centred at the means. SRQ-20 = Self-reporting Questionnaire; PHQ-9 = Patient Health Questionnaire-9; and GAD-7 = General Anxiety Disorder-7.

*p < 0.05. **p < 0.01.

### Baseline variables and PHQ-9 change score coefficients

A multiple linear regression investigating if age, gender, income, marital status and suicidal ideation level at baseline were associated with PHQ-9 change scores, while adjusting for baseline PHQ-9 scores, is reported in Table 4. The model was significant, with all variables accounting for 57% of the variance in PHQ-9 change scores. Age was found to be significantly associated with PHQ-9 change scores, suggesting that a higher PHQ-9 score decrease was observed for participants above 30 years compared to participants who were 30 years of age or younger. Suicidal ideation was significantly associated with PHQ-9 change scores, suggesting that a PHQ-9 score decrease was higher in participants who did not express suicidal ideation compared to those who did.

### Suicidal ideation

To further examine the finding that suicidal ideation level predicted PHQ-9 score change, a McNemar test was carried out to investigate if there was a difference in proportion in the suicidal ideation reported in item 9 of the PHQ-9 questionnaire (“Thoughts that you would be better off dead, or of hurting yourself in some way?”), between baseline and 4-weeks. Anyone scoring more than 0 (1–3) on the four-point scale were coded as a 1 to indicate the presence of suicidal ideation, and everyone scoring 0, were coded as 0 indicating absence of suicidal ideation. The McNemar test showed that the pre- and post-tests proportions were not different (χ^2^(1, *N* = 29) = 1.22, *p* = 0.25).

### Baseline variables and GAD-7 change score coefficients

A multiple linear regression investigating if age, gender, income, marital status and suicidal ideation level at baseline were associated with GAD-7 change scores, while adjusting for baseline GAD-7 scores is reported in Table 4. The model was significant, with all variables accounting for 46% of the variance in GAD-7 change scores. Gender was significantly associated with GAD-7 change scores, in which male participants observed a higher decrease in GAD-7 scores, compared to female participants. The association between income and GAD-7 scores was close to reaching statistical significance (*p* = 0.08), suggesting that participants with higher income (more than 10 000 KSh p/a) experienced a higher decrease in GAD-7 scores, compared to low income (below KSh. 10 000 p/a).

## Discussion

To our knowledge, this study is the first to pilot a PST intervention that is delivered by CHVs through a locally developed mobile app in Kenya. The study found preliminary evidence of clinically meaningful improvement in CMD symptoms.

The first and second objectives of the study were to evaluate the, acceptability of Inuka coaching for participants, and the difference between self-reported CMDs (SRQ-20 scores) across time (at baseline and 3 months). The findings suggested that the intervention was acceptable to participants with respect to their satisfcation rating of the mobile app and CHV. The findings also show that Inuka coaching was effective in reducing CMD symptoms. Our findings are in line with the Friendship Bench study, which found that a non-specialist worker delivered PST intervention was effective in reducing CMD symptoms, albeit through a more rigorous methodological design (Chibanda *et al*., 2011; Chibanda *et al*., 2016). Moreover, a Cochrane review on the effectiveness of interventions for mental, neurological and substance-abuse disorder that are delivered by non-specialist health workers in low- and middle-income countries found improved outcomes for depression, post-traumatic stress disorder and alcohol-use disorder (van Ginneken *et al*., 2013). While the adoption of digital technologies in the delivery of mental health services in low- and middle-income countries has largely focused on the training and education of non-specialist workers, more recently digital technologies have also been used to deliver mental health interventions (Naslund *et al*., 2019). A recent example comes from a trial in Indonesia, which found that internet-based behavioural activation with lay-counsellors support was more effective in reducing symptoms of depression compared with minimal online psychoeducation for depression without support, observing an effect size of 0.24 that was sustained at 3 and 6 months (Arjadi *et al*., 2018). Collectively, these results demonstrate promomising findings in relation to the feasibility and clinical effectiveness of internet-based mental health interventions that are supported by non-specialist workers.

The third objective of the study was to understand if specific baseline varibles, namely age, gender, income, marital status and, suicidal ideation level, were associated with CMD symptom improvement. The study found that having a higher income band (earnings of more than KSh 10 000 p/a) was associated with higher score reduction on the SRQ-20. Contrary to our findings, a systematic review on the predictors of recovery in people with CMDs in commmunity settings, in high-income countries found that socio-economic status did not have a direct impact on treatment outcome. However, the same review also found evidence that socioeconomic status had an impact on baseline depression scores, which is itself a predictor of outcome (Amati *et al*., 2017). The evidence on the impact of socioeconomic status on mental health outcomes between low- and middle-income countries and high-income countries, do not always align. While low socioeconomic status has been found to increase the risk of CMDs in high-income countries (Maselko, 2017), an inverse relationship between indicators of low socioeconomic status and CMDs was found in low- and middle-income countries (Lund, 2012; Lund, Breen, *et al*., 2010). The link between socioeconomic status, mental health and treatment outcomes appears to be complex and therefore requires further investigation.

Our study found that the male gender was associated with a higher GAD-7 score reduction, compared to females. In contrast, a systematic review on the predictors of treatment outcome for people with CMDs, found that being female was associated with positive treatment outcomes for patients receiving psychological therapies in high-income countries (Amati *et al*., 2017). Similarly, a study investigating the effectiveness of collaborative care for anxiety when compared to usual care found that women who received collaborative treatment showed clinical improvements, while no differences were found for men. The same study also found that women on the study, expressed more commitment to treatment, were in attendance of more psychotherapy sessions, completed more treatment modules, and viewed psychotherapy as more helpful, when compared to men. This may therefore explain why women showed clinical benefits, and men did not. The lack of engagement to mental health treatment in men, is also reflected in the wider literature around help-seeking behaviours, in which men were found to hold more negative attitude towards the utilisation of mental health services when compared to women (Addis and Mahalik, 2003; Mackenzie *et al*., 2006; Yousaf and Popat, 2015). A 2019 systematic review on the use of behaviour change techniques for improving mental health utilisation in men, found that ‘problem-solving’ and ‘behaviour change motivation’ were among the key processes identified to improve help-seeking attitudes, behaviours and intentions in men (Sagar-Ouriaghli *et al*., 2019). The application of these approaches in Inuka coaching may therefore explain why men in our study experienced a higher reduction of symptoms on the GAD-7. Furthermore, on average, men in our study spent 1.56 mins longer in the chat-session compared to women. Taken together, this may indicate that men engaged and clinically benefited from Inuka coaching more compared with women.

We also found that a higher-age range (over 30 years of age) compared to a lower age range (30 years of age or younger), was associated with a higher decline in PHQ-9 scores. The current evidence around the impact of age on digital mental health interventions outcomes is mixed. While some studies suggest that younger populations may benefit more from digital mental health interventions (Hampshire *et al*., 2015; Naslund *et al*., 2017), a meta-analytic review investigating if guided-internet-based interventions resulted in clinically relevant changes for clients, found that higher age was weakly associated with a better response to psychological interventions for depression (Karyotaki *et al*., 2018). Although the cause of our findings remain unclear, factors that both enhance engagement and increase with age, such as motivation or previous access to treatment, may moderate the association between age and treatment outcome (Karyotaki *et al*., 2018).

Lastly, we found that the absence of suicidal ideation predicted a higher score reduction on the SRQ-20 and PHQ-9 questionnaire, when compared to participants who reported suicidal ideation. In line with our findings, a study investigating if pre-treatment suicidal ideation predicted treatment outcome of a cognitive behavioural therapy (CBT) intervention for unipolar depression, found that pre-treatment suicidal ideation predicted higher post-treatment depression severity, after controlling for gender, age, number of attended therapy sessions, comorbidity and pre-treatment depression severity (von Brachel *et al*., 2019). These findings may explain why participants who reported suicidal ideation in our study experienced a lower reduction in PHQ-9 and SRQ-20 scores, compared to participants who did not report suicidal ideation. Our study also found that there were no differences in the presence of suicidal ideation on the PHQ-9 pre- and post-treatment. With a total of 26 participants who reported suicidal ideation, it is highly possible that the analysis was underpowered. However, there is some evidence to suggest that suicidal ideation may be a distinct phenomena, requiring targeted treatment to effectively address (Forkmann *et al*., 2013; Büscher *et al*., 2020; Torok *et al*., 2020). Nonetheless, treatment for depression is still beneficial for people with suicidal ideation (von Brachel *et al*., 2019). Future implementations of Inuka coaching should aim to employ appropriate protocols to assess clients with high suicidal ideation, to enable timely and targeted referrals under ‘Inuka coaching's’ referral principle.

There are a number of limitations that should be highlighted. First, the study was conducted as a single-arm, naturalistic, follow-up study in which no control condition was used. An RCT is therefore required to establish causal conclusions about the intervention. Another limitation pertains to only offering the intervention to people who were conversant in writing and speaking English. While the English language, one of two official languages of Kenya (the other being Swahili), was perceived as appropriate to address the country's multilingual population, Swahili remains the most commonly used language. The findings of our study therefore cannot be generalised to non-English speakers in this setting. The study did not formally examine the CHV's experience of delivering Inuka coaching. However, a brief qualitative exploration of the CHVs experience of delivering the intervention was explored in a previous phase of testing in another setting, that will be outlined in another paper. The current study formed one of many phases of ‘testing’, in an ongoing iterative process of developing, improving and testing the intervention (Curran *et al*., 2012). A final limitation concerns delivering the intervention in the health centre. While this approach was adopted for a range of pragmatic reasons (e.g. providing participants with assistance in using the app, reducing participation barriers for people who did not have access to a suitable/mobile device, and the internet etc.), this meant that participants were not able to take advantage of accessing the intervention from home, which was noted as a reasons for refusing participation in the study. In an attempt to mitigate some of the drawbacks of accessing the intervention from the health center, the study recompensed participants with KSh 300, so that they did not have to cover the costs of traveling to the health centre. Moreover, the CHVs did not know the identity of the participant seeking treatment, and were based in a different part of the health centre to avoid the risk of accidentally meeting. The intervention also took place in a health centre, which may have reduced the stigma associated with engaging in a mental health programme. While the main reason for not allocating mobile devices to participants stemmed from a lack of available resources, some benefits may have been derived from evaluating the intervention in a controlled environment. For example, participants were able to access assistance if they experienced technical problems that can be more prevelant in mobile apps that are in the Beta phase of development. This therefore allowed the research team to become aware of and resolve technical issue more rapidly, than would have been been possible if the intervention was accessed or delivered away from the health centre. Although it would not be possible to make inferences about the acceptability of using and delivering Inuka coaching outside of the health centre, refusal to take part in the study because the intervention could not be completed at home and due to clashes between working hours and the health centre's opening times, may suggest that some some individuals welcome the opportunity to use the intervention at home. Conversely, it is also possible that participants engaged in the study because the intervention took place in the health centre. Further research is required to examine how people wish to access digital mental health interventions in Kenya.

African has the fastest growing mobile network and smartphone access, leading more people in the continent to turn to their mobile devices to access health care advice (Hampshire *et al*., 2015; The Economist, 2015; GSMA, 2018). A study that surveyed 4500 young people across Ghana, Malawi, and South Africa, found that young people frequently used their mobile devices in the event of sickness, personal health crises, or in response to the health concerns of a friend or relative. Many respondents also used their mobile devices to contact other household members, friends, or neighbours to seek advice, recommendations, or support. These findings may imply that people in low-resource settings use mobile devices to seek informal support for their health-care needs in the absence of readily available services (Hampshire *et al*., 2015). Amidst increasing ubiquity of digital technologies in the region, Inuka coaching may therefore hold promise in addressing CMDs in the general population, and reach groups of people who are underserved in relation to mental health care.

### Future direction

Future research should robustly evaluate the Inuka coaching through an RCT to address the limitations of our methodological design. In-depth qualitative interviews should also be conducted to understand the clients’ and CHVs experience of using and delivering the intervention. The Inuka coaching programme should be offered in Swahili to increase the reach of the programme to non-English speakers in Kenya. Future evaluations should also investigate the feasibility of an improved version of the Inuka coaching app that has greater stability and can be used outside of the health centre, to enable the full benefits of adopting digital technologies in mental health care to be realised. The SSA region's very high care gap for mental health conditions warrants increasing the capacity for potentially cost-effective approaches such as task-shifting and digital technologies, in aid of supporting and expanding mental health care in low-resource settings. The use of digital technologies in healthcare is increasingly adopted in the global north. Not strengthening the capacity for digital mental health technologies and research in the low- and middle-income countries may further widen the digital divide in mental health care between the global north and the global south, and as a result restricting access to potentially efficacious and cost-saving mental health interventions (Peek, 2017).

## Footnotes

1 The health facilities (level III) offer standard primary care services, consisting of reproductive, maternal, neonatal and child health. Each facility is generally run by clinical officers and nurses and at least one doctor. Some of the services offered include, maternity in-patient services with a ward, curative services, laboratory services, pharmacy, TB clinics, diabetes and hypertension clinics, comprehensive care clinics for patients living with HIV, baby wellness clinics, antenatal and postnatal services and referrals to other facilities.

2 At the beginning of the study, the CHWs' use of their personal phones to deliver the intervention was not found to be feasible. Android phones were made available for the CHVs to use in the health centre.

3 The ‘Confounders’ questionnaire, and the ‘satisfaction with the Inuka coaching session and programme' questionnaire were not psychometrically validated. The data provided through these questionnaires were only used for descriptive purposes and were not used in the main analysis.

## Supplementary material

For supplementary material accompanying this paper visit http://dx.doi.org/10.1017/gmh.2021.3. click here to view supplementary material
