# A community health volunteer delivered problem-solving therapy mobile application based on the Friendship Bench ‘Inuka Coaching’ in Kenya: A pilot cohort study – CORRIGENDUM

**DOI:** 10.1017/gmh.2023.54

**Published:** 2023-09-15

**Authors:** Asmae Doukani, Robin van Dalen, Hristo Valev, Annie Njenga, Francesco Sera, Dixon Chibanda

The authors apologise that within the table provided for Table 4 some of the confidence intervals are incorrect, as well as some inconsistency in the way they have been reported

The table has been corrected as below:

**Table 4.** Summary of a Multiple Linear Regression Model for Variables Predicting SRQ-20, PHQ-9 and GAD-7 change scores

	SRQ-20			PHQ-9			GAD-7		
	B	SE B	CI 95	B	SE B	CI 95	B	SE B	CI 95
Age	–0.09	1.41	–2.676 to 2.854	–5.89	2.26	1.460 to 10.323*	1.11	2.80	–4.371 to 6.590
Gender	–1.71	1.39	–4.440 to 1.021	–1.78	2.72	–7.107 to 3.538	–7.52	3.65	–14.678 to -0.367*
Income	–3.17	1.53	–6.162 to -0.180*	–0.37	2.44	–5.166 to 4.417	–5.68	3.13	–11.825 to 0.463
Marital status	0.43	1.34	–2.208 to 3.062	–0.28	2.14	–4.468 to 3.911	–0.62	2.81	–6.130 to 4.880
Suicidal ideation	4.44	1.46	1.584 to 7.288**	4.63	2.19	0.335 to 8.923*	2.85	2.88	–2.794 to 8.494
	R^2^ = 0.25; F for change in R^2^= 2.87*			R^2^ = 0.57; F for change in R^2^= 4.57**			R^2^ = 0.46; F for change in R^2^= 2.98*		

*Note:* Age was centred at the means. SRQ-20= Self-reporting Questionnaire-20; PHQ-9= Patient Health Questionnaire-9, and GAD-7= General Anxiety Disorder-7.

* *p* < .05.

** *p* < .01.
