# Supplementary material for: A community health volunteer delivered problem-solving therapy mobile application based on the Friendship Bench ‘Inuka Coaching’ in Kenya: A pilot cohort study
Source: Glob Ment Health (Camb). 2021 Mar 10;8:e9. doi: 10.1017/gmh.2021.3 (PMC8127638; doi:10.1017/gmh.2021.3)
Supplement: Supplementary file 1 [file S2054425121000030sup001.docx]

**Supplementary Material**

| **Supplementary Table 1**  Average time (in minutes) spent per session by gender | | | |
| --- | --- | --- | --- |
| **Session** | **Average time (mins) spent in session (n=60)** | | |
|  | *Male* | *Female* | *Overall sample* |
| Session 1 | 54.73 | 55.21 | 55.03 |
| Session 2 | 47.09 | 45.39 | 46.02 |
| Session 3 | 43.14 | 39.87 | 40.43 |
| Session 4 | 28.41 | 26.68 | 27.52 |
| Mean | 43.34 | 41.78 | 41.79 |

| **Supplementary Table 2**  Participants average rating on a scale of 1-4 of satisfaction in relation to CHV and the Inuka program. | | |
| --- | --- | --- |
| Survey questions (n=47) | CHV | Program |
| 1. How would you rate the quality of the session/program you received? | 3.67 | 3.63 |
| 2. Did you get the kind of session/program you wanted? | 3 | 3.70 |
| 3. To what extent has the session/program met your needs? | 3.39 | 3.39 |
| 4. How satisfied are you with the amount of help you received/ during the program? | 4 | 3.96 |
| 5. Has the session/program you received helped you to deal more effectively with your problems? | 3.78 | 3 |
| 6. In an overall, general sense, how satisfied are you with the session/ program you received? | 3.96 | 4 |
| 7. If a friend were in need of similar help, would you recommend our program to him or her? | n/a | 3.85 |
| 8. If you were to seek help again, would you come back to our program? | n/a | 3.93 |
